# Associations of SARS-CoV-2 cycle threshold values with age, gender, sample collection setting, and pandemic period

**DOI:** 10.1590/S1678-9946202365053

**Published:** 2023-10-20

**Authors:** Fernando Franco-Miraglia, Beatriz Martins-Freitas, André Mario Doi, Rubia Anita Ferraz Santana, João Renato Rebello Pinho, Vivian I. Avelino-Silva

**Affiliations:** 1 Hospital Israelita Albert Einstein, Faculdade Israelita de Ciências da Saúde Albert Einstein, São Paulo, São Paulo, Brazil; 2 Hospital Israelita Albert Einstein, São Paulo, São Paulo, Brazil; 3 Universidade de São Paulo, Faculdade de Medicina, Departamento de Moléstias Infecciosas e Parasitárias, São Paulo, São Paulo, Brazil

**Keywords:** SARS-CoV-2, COVID-19, Reverse transcriptase polymerase chain reaction, SARS-CoV-2 variants, Viral load, COVID-19 vaccines, Demographic factors

## Abstract

Cycle threshold (Ct) values in COVID-19 reverse-transcription polymerase chain reaction (RT-PCR) tests estimate the viral load in biological samples. Studies have investigated variables associated with SARS-CoV-2 viral load, aiming to identify factors associated with higher transmissibility. Using the results from tests performed between May/2020-July/2022 obtained from the database of a referent hospital in Sao Paulo, Brazil, we investigated associations between Ct values and patient’s age, gender, sample collection setting and pandemic period according to the predominant SARS-CoV-2 variant locally. We also examined variations in Ct values, COVID-19 incidence, mortality, and vaccination coverage over time. The study sample included 42,741 tests. Gender was not significantly associated with Ct values. Age, sample collection setting and the pandemic period were significantly associated with Ct values even after adjustment to the multivariable model. Results showed lower Ct values in older groups, during the Gamma and Delta periods, and in samples collected in emergency units; and higher Ct values in children under 10 years old, home-based tests, during the Omicron period. We found evidence of a linear trend in the association between age and Ct values, with Ct values decreasing as age increases. We found no clear temporal associations between Ct values and local indicators of COVID-19 incidence, mortality, or vaccination between February/2020-November/2022. Our findings suggest that SARS-CoV-2 Ct values, a proxy for viral load and transmissibility, can be influenced by demographic and epidemiological variables.

## INTRODUCTION

Reverse Transcription Polymerase Chain Reaction (RT-PCR) nucleic acid amplification is the gold standard diagnostic test to document the presence of SARS-CoV-2 in respiratory samples^1^. RT-PCR has been extensively used from the onset of the COVID-19 pandemic to detect SARS-CoV-2 in both symptomatic and asymptomatic individuals. However, most RT-PCR assays provide not only qualitative but also quantitative results, estimating the viral load in a given sample as a function of the cycle threshold (Ct) value^2-4^. Ct measures the number of PCR cycles needed for the fluorescence level in a sample, which increases proportionally to the amount of amplified RNA, to surpass the cutoff for positivity; thus, the higher the Ct, the lower the expected RNA amount in a sample^5^.

Although Ct is not a direct measure of the amount of viable and infecting virus copies in a sample, prior studies indicate a relationship between lower Ct values and higher probability of viral isolation in cell cultures^6,7^. Since identifying and isolating potential SARS-CoV-2 spreaders is an important strategy to control virus transmission, studies have adopted Ct values as proxies for viral loads to identify features that confer higher or lower infectivity to a patient by analyzing associations between Ct values and clinical or demographic characteristics^1^.

To better understand the dynamics and determinants of SARS-CoV-2 Ct values, studies have investigated its association with demographic factors such as age, gender, and race. Thus far, results concerning the effects of age on Ct values have been inconsistent^4,8-11^. Research exploring associations between Ct values and clinical presentation suggests that higher viral loads may be associated with an increased risk of intubation, overall severity, and mortality; yet, other studies have shown different results^8,12-14^.

While the SARS-CoV-2 Ct dynamics in asymptomatic or pre-symptomatic patients are poorly understood, Ct values typically vary over the course of symptomatic COVID-19 in each individual^15^. Both longitudinal and cross-sectional studies have observed lower values in the first days after symptoms onset, followed by a progressive increase in subsequent days^8,9,15-18^. In studies that do not involve longitudinal follow-up of participants, the sample collection setting can be used as a proxy of clinical presentation, sometimes enabling assumptions about the presence of symptoms and their duration. Samples collected for screening purposes, for example, such as those obtained from athletes and company employees, are presumably more often collected from asymptomatic or pre-symptomatic individuals. Conversely, patients tested in hospitals or emergency services are more likely symptomatic and in the initial phases of disease onset, while those tested in outpatient laboratory facilities presumably had less intense symptoms. On the other hand, patients tested at home are more likely to undergo sample collection several days after symptoms onset due to delays in test scheduling. Hence, the location and sample collection setting could influence Ct values.

Finally, some studies suggest that Ct values can show population-level variations over different periods of the pandemic, with lower mean values coinciding with periods of rising spread, such as those seen during the upsurge of novel SARS-CoV-2 variants^1,15^. Multiple variants have been identified since the beginning of the pandemic, with different pathogenicity and infectivity profiles^19,20^. In Brazil, predominant lineages included B1.195, B.1.1.128 and B1.1.33^21-23^. P.2 variant may have first appeared in the country in July 2020, and P.1 surge probably occurred in December of the same year. Both P.1 and P.2 seem to be derived from B.1.1.28. P.2 spread throughout the country between September 2020 and January 2021, whereas P.1 saw even faster dissemination, covering the entire Brazilian territory between January and February 2021^23^. Gamma, as P.1 was latter designated, became the most frequently detected variant among all SARS-CoV-2 strains in RT-PCR tests in Sao Paulo State and across Brazil by February 2021; its spread was accompanied by an important surge in the number of COVID-19 cases, corresponding to the “second wave” of the pandemic in the country^23-25^. By August 2021, when reported deaths and cases were declining, Delta had replaced Gamma as the most prominent variant in Brazil^26^. Finally, the Omicron variant, first reported in South Africa, surpassed the detection rate of the Delta variant in Sao Paulo around November 2021, and was associated with a fast increase in the number of COVID-19 cases^24,27,28^.

In this study, we used information collected from the database of a reference laboratory in Sao Paulo, Brazil (23° 32’ 56”S, 46° 38’ 20”W) to investigate associations between Ct values and patient’s age, gender, sample collection setting and pandemic period, categorized according to the predominant SARS-CoV-2 variant locally. We also explored variations in Ct values, incidence of COVID-19 cases, mortality, and vaccination coverage over time.

## MATERIALS AND METHODS

This cross-sectional study included data from patients with SARS-CoV-2 infection confirmed by a positive RT-PCR test performed in nasal and/or oral cavity swabs at the Hospital Israelita Albert Einstein laboratory. This tertiary hospital has a reference laboratory that supports 17 inpatient and outpatient facilities located in 11 different municipalities in the Sao Paulo State with diagnostic resources. Participants included in the study underwent RT-PCR testing as requested by their medical providers, without additional tests for the purpose of this research. Qualified personnel processed the samples according to institution’s standards and the test manufacturer’s instructions, storing the test results, including Ct values, in a centralized database along with demographic information. We initially selected all patients who underwent SARS-CoV-2 testing from May 14, 2020 to July 26, 2022. From this initial sample, we used Python (version 3.9.7, Python Software Foundation, Delaware, USA) and libraries pandas 1.3.3 and NumPy 1.20.3 to identify all patients whose test had been done using the Cobas-6800 System and Cobas^®^ SARS-CoV-2 test (Roche Diagnostics, Basel, Switzerland). For each unique patient, we maintained only the first test in the analysis to eliminate test results that could correspond to recurrent testing, and to focus on early disease onset.

Ct measurements are influenced by factors such as the source and type of specimen, collection technique, and technical characteristics of the assay^3^. Thus, we opted for restricting our analysis to Ct values generated with a single RT-PCR equipment to avoid potential biases generated by different manufacturers and models. We selected the Cobas^®^ SARS-CoV-2 assay because it had the highest and most stable volume of results throughout the study period. The assay generates two Ct values for each sample: one for gene E and another for ORF1a/b. Only the results for gene ORF1a/b were included in this study.

We compared Ct values across different sample collection settings categorized as those made primarily with screening purposes (athletes and company employees); and those collected in laboratory units; emergency units; hospitals; outpatient clinics; and home-based tests. Our analysis included these categories to explore differences in Ct values associated with the collection setting, which could indirectly indicate the presence/severity of symptoms and time since their onset if applicable.

Participant characteristics are presented as counts and percentages for categorical variables, and medians and interquartile ranges (IQR) for numerical variables. Age is presented both as a numeric variable (years) and as a categorical variable (decade). We used data from the SARS-OMICs database, which includes test results from Hospital Israelita Albert Einstein, to define four pandemic periods according to the predominant SARS-CoV-2 variant: October 2020 to December 2020 (pre-Gamma); January to July 2021 (Gamma); August 2021 to October 2021 (Delta); and November 2021 to September 2022 (Omicron)^24^.

Associations between Ct values and patient’s age, gender, sample collection setting and pandemic period were calculated using descriptive analysis, as well as univariable and multivariable linear regression models with robust standard errors and a 0.05 significance level. In the univariable models, we present coefficients (effect estimates) for each category of the independent variables compared to a referent category, along with its 95% confidence intervals. The multivariable model included all variables to explore the association of each independent variable adjusted for the remaining covariates studied. We used a linear trend test to assess if Ct values varied linearly across age groups.

Finally, we evaluated the relation between Ct values, COVID-19 incidence, mortality, and vaccination coverage over time between February 2020 and November 2022, using graphical methods and health indicators made available online by the state health department^29,30^. COVID-19 incidence and mortality in the Sao Paulo State (total population of 46,649,132 residents) were presented as frequencies/counts per month^31^. Vaccination coverage in the Sao Paulo extended metropolitan area was described as cumulative percentage per month. The state’s extended metropolitan region includes ten municipalities: Sao Paulo, Cotia, Franco da Rocha, Guarulhos, Mogi das Cruzes, Osasco, Santana do Parnaiba, Sao Bernardo do Campo, Suzano, and Taboao da Serra, with an estimated population of 16,970,273 inhabitants^31^.

Statistical package Stata (version 15.1, StataCorp LLC, Texas, USA) and Microsoft^®^ Excel (version 16.68, Microsoft Corporation, Washington, USA) were used for all analyses.

The institution’s ethics committee reviewed and approved the study with exemption of informed consent (CAAE 34882620.7.0000.0071, SGPP 4280-20). We kept all identifiable patient information confidential throughout the study.

## RESULTS

A total of 1,088,682 RT-PCR tests were collected between May 2020 and July 2022 using the Cobas-6800 System and Cobas^®^ SARS-CoV-2 test and registered in the institution’s database. Of these, we excluded 933,671 tests with a negative result and/or with a Ct value higher than 38 (n=155,011); 62,764 positive results without available corresponding Ct values (n=92,247); 163 tests collected in settings outside the study categories (n=92,084); 45,941 results for gene E (n=46,143); 2,202 test repetitions for the same patient (n=43,941); one test with inconsistent Ct value (n=43,940); four tests from patients younger than one-year-old inconsistently categorized as athletes or employees (n=43,946); and 1,194 results from small municipalities where vaccination and testing dynamics could differ from those in the Sao Paulo extended metropolitan region (n=42,741).

Hence, the final sample included 42,741 tests. Table 1 summarizes the demographic characteristics of study participants, sample collection settings, and pandemic periods. Most participants were female (54.7%), aged 20 to 59 years old (66.5%), and tested during the Omicron (49.3%) or pre-Gamma (39.3%) variant predominance. Laboratories were the most frequent sample collection setting (24.4%), followed by athletes and employees (20.7%), outpatient clinics (18.5%) and emergency units (17.8%).

**Figure 1:**
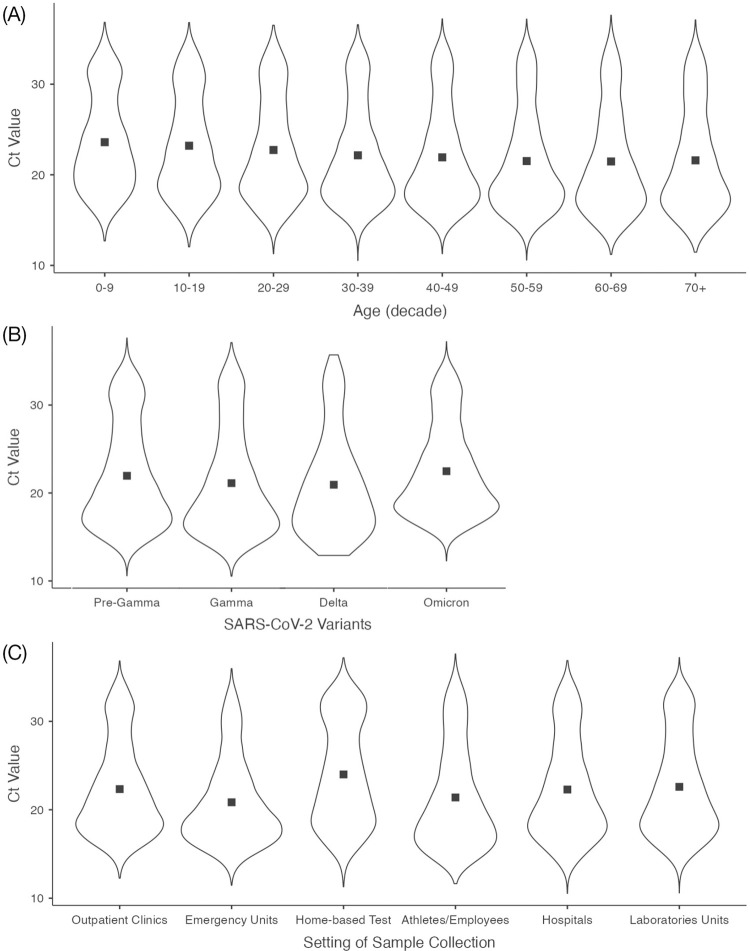
(A) Cycle threshold values distributed by age groups. Squares indicate mean values; (B) Cycle threshold values distributed by pandemic period. Squares indicate mean values; (C) Cycle threshold values distributed by sample collection setting. Squares indicate mean values.

The 50-59 and 60-69 age groups (21.5), emergency units (20.8), athletes and employees (21.4) and Delta variant period (20.9) showed the lowest mean Ct values. In turn, age group 0-9 years (23.6), home-based tests (24.0) and Omicron period (22.5) presented the highest mean Ct values (Table 1). Figures 1A, 1B and 1C show the distributions of Ct values by age group, pandemic period and sample collection setting, respectively.

**Table 1:** Characteristics of study participants.

Variables	Distribution	Median Ct (IQR)	Mean Ct
**All participants (%)**	42,741 (100)	20.7 (17.8-25.9)	22.1
**Median Age (IQR)**	41 (29.0-56.0)	-	
<10 years old (%)	2,107 (4.9)	22.6 (19.4-27.6)	23.6
10-19 years old (%)	3,223 (7.5)	22.3 (18.7-27.5)	23.2
20-29 years old (%)	5,450 (12.7)	21.4 (18.3-27.0)	22.7
30-39 years old (%)	8,885 (20.7)	20.8 (17.9-25.6)	22.2
40-49 years old (%)	8,361 (19.5)	20.4 (17.7-25.4)	21.9
50-59 years old (%)	5,839 (13.6)	19.9 (17.3-24.9)	21.5
60-69 years old (%)	4,767 (11.1)	20.0 (17.0-25.2)	21.5
70+ years old (%)	4,109 (9.6)	20.1 (17.1-25.5)	21.6
**Gender (%)** ^**a**^			
Female	23,417 (54.7)	20.7 (17.8-25.8)	22.1
Male	19,276 (45.1)	20.7 (17.7-26.0)	22.1
**Sample collection setting (%)** ^**b**^			
Emergency Units	7,621 (17.8)	19.5 (17.2-23.5)	20.8
Athletes/Employees	8,886 (20.7)	19.8 (16.9-25.2)	21.4
Laboratory Units	10,442 (24.4)	21.3 (18.1-26.7)	22.6
Hospitals	3,076 (7.2)	20.9 (17.9-26.3)	22.3
Outpatient Clinics	7,928 (18.5)	21.1 (18.2-25.7)	22.3
Home-based Test	4,762 (11.1)	23.1 (19.0-29.3)	24.0
**Pandemic period (%)**			
Pre-Gamma	16,809 (39.3)	20.4 (17.2-26.3)	22.0
Gamma	4,653 (10.8)	19.4 (16.5-24.8)	21.1
Delta	194 (0.4)	19.4 (16.4-23.9)	20.9
Omicron	21,085 (49.3)	21.2 (18.4-25.8)	22.5

IQR = interquartile range; Ct = cycle threshold; ^a^missing for 48 participants; ^b^missing for 26 participants.

Table 2 summarizes the results from the univariable and multivariable linear regression models. We found no statistically significant differences between Ct values of male and female participants. Both univariable and multivariable analyses showed a consistent, statistically significant association between age and Ct values, with decreasing Ct values associated with older age. Accordingly, the multivariable model indicated a statistically significant linear trend in the association between age categories and Ct values (p<0.0001). Sample collection setting was also significantly associated with Ct values, with the lowest Ct values observed in samples collected in emergency units. Samples from home-based tests had, on average, a 3.10-unit higher Ct value (95% confidence interval 2.90 to 3.30, p<0.001) adjusted for age, gender, and pandemic period.

**Table 2:** Univariable and multivariable linear regression models for the associations between age group, gender, sample collection setting, and pandemic period with SARS-CoV-2 RT-PCR cycle thresholds.

Variables	Univariable analysis Coefficient (95% CI)	Multivariable analysis Coefficient (95% CI)	Multivariable analysis p-values
**Age group**			
<10 years old	0 (reference)	0 (reference)	-
10-19 years old	−0.38 (−0.68 to -0.88)	−0.34 (−0.64 to −0.05)	0.022
20-29 years old	−0.86 (−1.13 to -0.58)	−0.87 (−1,15 to −0.60)	<0.001
30-39 years old	−1.44 (−1.70 to -1.18)	−1.38 (−1.64 to −1.12)	<0.001
40-49 years old	−1.67 (−1.93 to -1.41)	−1.56 (−1.82 to −1.30)	<0.001
50-59 years old	−2.08 (−2.35 to -1.80)	−1.88 (−2.15 to −1.60)	<0.001
60-69 years old	−2.12 (−2.40 to -1.84)	−1.83 (−2.12 to −1.54)	<0.001
70+ years old	−2.00 (−2.29 to -1.71)	−1.79 (−2.09 to −1.48)	<0.001
**Gender**			
Female	0 (reference)	0 (reference)	-
Male	-0.03 (-0.13 to 0.07)	-0.07 (-0.17 to 0.03)	0.169
**Sample collection setting**			
Emergency Units	0 (reference)	0 (reference)	-
Athletes/Employees	0.54 (0.37 to 0.71)	1.24 (1.04 to 1.44)	<0.001
Laboratory Units	1.74 (1.58 to 1.90)	1.76 (1.60 to 1.92)	<0.001
Hospitals	1.44 (1.21 to 1.67)	1.52 (1.29 to 1.75)	<0.001
Outpatient Clinics	1.49 (1.32 to 1.66)	1.41 (1.24 to 1.58)	<0.001
Home-based Test	3.15 (2.95 to 3.34)	3.10 (2.90 to 3.30)	<0.001
**Period according to predominant variant**			
Pre-Gamma	0 (reference)	0 (reference)	-
Gamma	−0.83 (−1.01 to −0.65)	−0.84 (−1.02 to −0.66)	<0.001
Delta	−1.02 (−1.79 to −0.24)	−1.36 (−2.13 to −0.59)	<0.001
Omicron	0.51 (0.40 to 0.63)	0.18 (0.05 to 0.30)	0.004

CI = confidence intervals.

Finally, the pandemic period showed statistically significant association with Ct values, with higher Ct values found in samples from the Omicron predominance period (Table 2).

**Figure 2:**
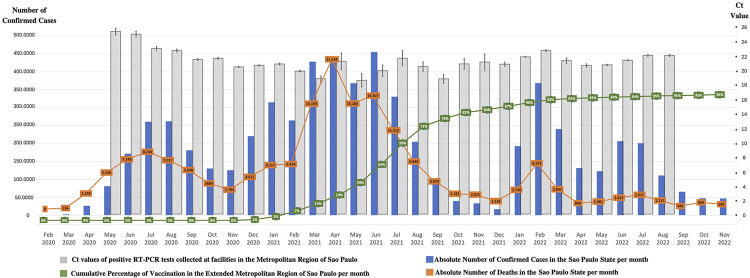
Cycle threshold values, COVID-19 incidence and mortality, and vaccination coverage over time.

Figure 2 presents a graphical evaluation of Ct results over time along with indicators of COVID-19 incidence (absolute number of cases per month), mortality (absolute number of deaths per month), and vaccination coverage (cumulative percentage per month). We observed no clear changes in Ct values according to these factors, neither a consistent trend over time; however, we observed a noticeable decrease in the mortality-to-cases ratio following the increase in vaccination coverage.

## DISCUSSION

This study investigated associations between age, gender, sample collection setting, and pandemic period defined by the predominant SARS-CoV-2 variant locally and Ct values, used as a proxy of viral loads in respiratory secretions. Age, sample collection setting and pandemic period were significantly associated with Ct values even after adjustment in a multivariable model. We observed lower Ct values in samples from participants tested for screening purposes (athletes and company employees) and in emergency units, possibly because these individuals are likely pre-symptomatic or seeking care early in the disease onset, as seen in other studies^15-18^. Higher Ct values among participants tested at home could also be explained by this phenomenon, given that these tests are usually scheduled after symptoms onset, leading to delays compared with other testing modalities. Different disease presentation patterns may play a role in these results, as individuals undergoing testing in emergency units are expected to be more symptomatic than patients in other settings.

Previous studies have shown conflicting results concerning the correlation between SARS-CoV-2 viral load and disease severity. While a lower Ct value could reflect higher viral replication and direct tissue damage, tests done at later timepoints in the disease course—in patients with escalating symptoms and exacerbated inflammatory response—could also show higher Ct values^8,12,32^. Shah et al.’s systematic review and meta-analysis showed that hospitalized patients with lower Ct values had a higher mortality risk and disease severity^33^. Another study found an association between higher Ct values and increased odds of severe presentation^8^. Finally, a third study failed to find statistically significant associations between Ct values and poor outcomes^34^.

As observed in other publications, we found no consistent association between gender and Ct values^8,35^; however, age and Ct values showed a statistically significant association, suggesting lower SARS-CoV-2 viral loads in younger age groups. Observed in both the univariable and multivariable analyses, this finding was reinforced by a significant linear trend test. The effect of age on Ct values is not well established in the literature^8,36^. Viral loads analysis of 260 patients younger than 21 years old found similar Ct values across age groups, regardless of symptoms onset^9^. Another study with 405 participants, all within the first five days after symptoms onset, found no associations between age and Ct values^10^. Conversely, a German research including 3,303 COVID-19 cases found statistically significant differences between some age groups, albeit limited by the use of different assays, small number of pediatric patients, and absence of clinical data^4^. A larger study including 21,831 participants found lower Ct values in young adults compared with other age groups, but these effects lost significance after adjustment for symptoms and number of positive genes^11^. Associations between age and Ct values could be related to behavioral factors, such as health care-seeking patterns, or age-associated differences in immunological and physiological characteristics, including clinical presentation. Understanding the mechanisms underlying such effects would require further studies based on more detailed clinical data.

Prior research suggests that a decrease in average Ct values in a population may occur during or preceding an increase in the incidence of COVID-19 cases, a pattern that could be used to predict changes in disease incidence over time^1,11,15^. Such variation could result from the introduction and dissemination of a new variant for which most individuals have limited immunity, thus presenting with higher viral load following infection. Moreover, lower Ct values in a population are presumably associated with higher transmissibility and a rise in disease incidence. Evidence for this hypothesis comes from a study in which Ct values were inversely correlated with outbreak indicators including daily changes in COVID-19 percent positivity, transmission rates, and hospitalizations^37^. Our analysis found no clear association between average Ct values and the emergence of new variants. By selecting only the first RT-PCR test for patients tested more than once, we excluded individuals at later phases of the disease course whose augmented prevalence in populations evaluated by cross-sectional sampling during a waning epidemic could potentially increase average Ct values^15^.

The changes in average Ct values over time observed could be partially related to SARS-CoV-2 variant local predominance, vaccination coverage, as well as behavioral and testing dynamics. For instance, point-of-care rapid tests became increasingly popular in Brazil over time and their widespread use may have resulted in those with early and mild symptoms being tested at home and seeking medical care only when presenting severe symptoms^38,39^. We hypothesized that a rise in vaccination coverage could lead individuals to being less fearful of severe disease complications and therefore being less prone to seek medical care early in the disease course, with both behaviors leading to higher average Ct values over time. Nevertheless, we found no clear evidence of such trends.

Our study had a few limitations. The cross-sectional analysis of retrospective data limited our ability to draw causal inferences. We lacked access to clinical information including presence of symptoms, time since symptoms onset, vaccination status, or comorbidities. As we restricted our analysis to tests done in the Cobas-6800 platform and Cobas^®^ SARS-CoV-2 test, the Delta variant period had a smaller number of samples. Our dataset included tests performed in a single institution, and even though they comprised samples collected in ten different cities in the Sao Paulo State, generalizability to other populations or settings may be limited. Finally, the absence of individual-level information on the infecting variant lead us to estimate the most likely predominance using external sources, which could result in residual confounding.

Despite these limitations, the strengths of this study include its large volume of samples, obtained from a reference laboratory located in one of the most affected countries worldwide, and analyzed by only one type of RT-PCR assay. The lower Ct values observed in samples from older adults during the Gamma and Delta variants predominance periods, and in samples collected at emergency units is relevant for developing control strategies against disease spread aimed at individuals with characteristics associated with higher viral loads and, thus, higher risk of virus transmission.

## CONCLUSION

Our findings point to a significant association between SARS-CoV-2 Ct values and age, pandemic period and sample collection setting, with lower Ct values associated with older age groups, the Gamma and Delta periods, and samples collected in emergency units. We failed to find statistically significant associations between Ct values and gender. Moreover, we found no clear association between average SARS-CoV-2 Ct values and COVID-19 incidence, mortality, or vaccination coverage. Our results suggest that SARS-CoV-2 Ct values, a proxy for viral load and transmissibility, can be influenced by some demographic and epidemiological variables. Identifying factors associated with higher COVID-19 transmissibility as well as other infections could help to develop targeted prevention strategies.
